# A Case of Rice Body Synovitis of the Knee Joint

**DOI:** 10.1111/os.13195

**Published:** 2022-03-16

**Authors:** Zhao Haibo, Wang Tianrui, Song Wenlian, Sun Shenjie, Li Chunpu, Zhao Xia, Yu Tengbo, Zhang Yingze

**Affiliations:** ^1^ Qingdao University Qingdao China; ^2^ Orthopedics Department of the Affiliated Hospital of Qingdao University Qingdao China; ^3^ Trauma Emergency Center of the Third Affiliated Hospital of Hebei Medical University Shijiazhuang China

**Keywords:** Knee joint, MRI, Synovitis

## Abstract

**Background:**

Rice body synovitis (RBS) is a rare disease. It is prone to be developed due to rheumatoid disorder or tuberculosis infection. Additional infectious arthritis (non‐tuberculous mycobacterial infection and fungal infection), juvenile arthritis, the onset of adult Still's disease, systemic lupus erythematosus (SLE), seronegative arthritis, and non‐specific arthritis. The clinical imaging, histopathological features, and surgical treatment process of a patient were documented combined with literature. Furthermore, differentiation was performed with additional synovitis diseases so that the cognition of synovitis could be enhanced for clinical reference.

**Case Presentation:**

The present study reported a 50‐year‐old female patient who suffered from intermittent left knee pain with limited movement for 9  years. The conditions were aggravated after long‐term standing or walking and remitted after taking a rest, accompanied by noose and jamming. The specialist range of motion (ROM) examinations of the left knee revealed: 30° ‐ 0° ‐ 110° and left McMurray sign (+). Plain MRI scanning revealed that in the left knee cavity and the popliteal fossa area, a large number of low signals on free rice‐like bodies were visible inside and the lower femur and the upper tibia exhibited abnormally high signals of patchy lipography. Surgical exploration revealed numerous rice‐like free bodies in the suprapatellar bursa, the intercondylar fossa, and the posterior articular capsule. The patient presently has resolution of symptoms after surgical treatment.

**Conclusions:**

The RBS of the knee joint is very rare in the clinic. As MRI examination can provide valuable information, clinicians should actively perform MRI examination. Once the disease is diagnosed by examination, surgery is the optimal treatment.

## Graphical abstract

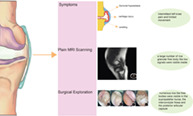

The clinical, imaging, histopathological features, and surgical treatment process of the patient were documented combined with literature. As MRI examination can provide valuable information, clinicians should actively perform MRI. Once the disease is diagnosed by examination, surgery is the optimal treatment.

## Introduction

Rice body (RB) has been reported as an intraarticular proteinaceous mass infected by tuberculosis before^1^, ^2^. However, few reports have been documented on RB later on, and the RB formation has been linked to infectious arthritis (tuberculosis and atypical mycobacterium infection), rheumatoid arthritis, traumatic arthritis, seronegative arthritis, juvenile arthritis, osteoarthritis, and chronic bursitis^3^, ^4^, ^5^. The present report described a 50‐year‐old female patient who had developed clinical, radiological, and histopathological features. The presence of RB was in the left knee joint while the patient reported no history of inflammation. These findings allow us to have a better understanding of knee RB and provide a reference for the diagnosis and treatment of patients.

## Case Report

We reported the case of a 50‐year‐old female, who presented at the hospital for intermittent pain in the left knee joint accompanied by movement limitation for 9  years and aggravated 1  year earlier. The disease had started insidiously in the absence of an obvious cause. The symptoms were aggravated after standing or walking for a long time but remitted after rest, accompanied by noose and jamming. The patient subsequently underwent a digital radiography (DR) examination and received conservative treatment in a local hospital. Unexpectedly, her pain worsened so she sought medical care in our hospital. The patient had no previous history, personal history, family history of hereditary tendencies or infectious diseases.

The general conditions were normal through physical examinations. The specialist examinations presented normal skin color in the left knee joint. No subcutaneous congestion, venous irritation, or sinus tract was revealed and the skin temperature was normal. Tenderness was reported in the interarticular space of the left knee. The floating patella test of the left knee joint was negative (−). The dorsalis pedis artery was palpable and the skin sensation was normal. Range of motion of the left knee revealed: 30° ~ 0° ~ 110°, left McMurray sign (+), anterior and posterior drawer tests (−), Lachman test (−), lateral stress test (+), patellofemoral grinding test of the right knee (−), and tibiofemoral grinding test of both knees (−). The muscle strength of both knee extensors and flexors was at V level. No obvious abnormality was observed in other limbs. The preliminary diagnosis was a loose body of the knee joint (left lateral).

**Fig. 1:**
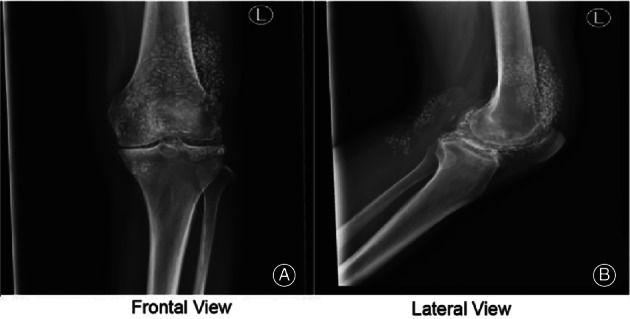
(A) DR of the left knee joint: narrowness in the interarticular space of the left knee joint was observed. Bone hyperplasia of different degrees was visible at the articular surface edge and the intercondylar eminence turned sharp. (B) Multiple speckled dense shadows were visualized around the area.

**Fig. 2:**
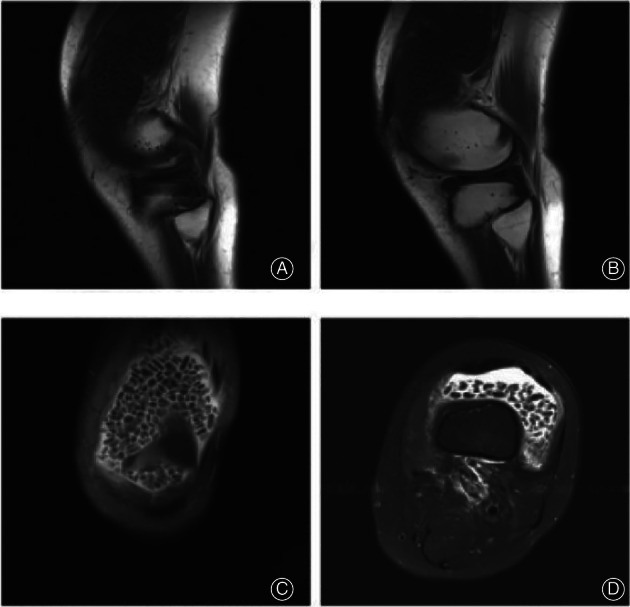
(A, B) Plain MRI scanning of the left knee joint. (A) MRT1WI sagittal knee joint. (B) MRT2WI sagittal knee joint. (C) MRT2 coronal knee joint. (D) MTR2 transverse knee joint.

Imaging examinations included an anteroposterior DR and an MRI plain scan of the left knee joint (Figs 1, 2). After a case discussion in the department, the patient was introduced to surgical treatment. Preoperative chest CT plain scan findings revealed several scattered stripes and minor patchy shadows in both lungs, with unclear boundaries. The patient was therefore diagnosed with probable chronic inflammation in both lungs. Laboratory examinations indicated a mild increase of leukocytes in urine. The levels of total cholesterol, apolipoprotein A1, apolipoprotein B, low density lipoprotein, and free fatty acid in the blood were increased slightly. The lymphocyte count and percentage were decreased whereas the neutrophil percentage was elevated. The rest were normal.

**Fig. 3:**
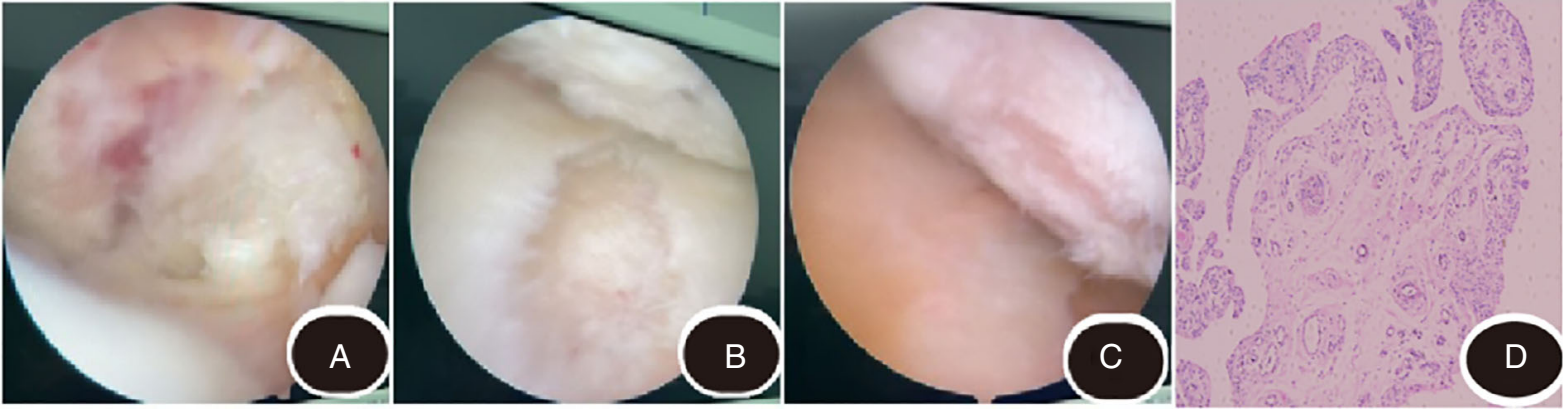
Intraoperatively, the patellar articular surface of the knee joint was roughly distributed and degenerated seriously. (A, B) The medial femoral condyle and the cartilage of the corresponding tibial articular surface were partially injured. More synovial folds were revealed in the lateral intersulcus. (C) Synovial hyperplasia was found in the articular cavity. (D) Pathological section of the synovial membrane of right shoulder joint. Microscopically, it is a degenerative infarct, red stained nodule with no structure, a small amount of lymphocyte infiltration in the interstitium.

**Fig. 4:**
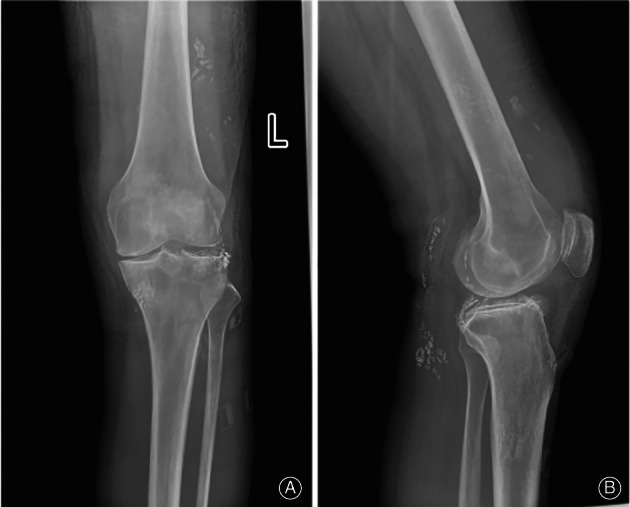
The anteroposterior (A) and lateral digital (B) radiography of the left knee. The medial space of the left knee joint narrowed, and the edges of the articular surface showed different degrees of bone hyperplasia, and the intercondylar bulge mutations. Multiple spot‐like high‐density shadows can be seen around.

Arthroscopic surgical exploration was performed 2  days later and intraoperative samples were delivered for pathological examination. The procedure was “arthroscopic knee joint synovectomy + arthroscopic removal of the knee joint free body + arthroscopic resection of the knee lesions + arthroscopic knee joint examination + therapeutic drug injection for the knee joint” (Fig. 3). The detailed procedures, intraoperative findings, and treatment were as follows:

After successful anesthesia, the patient was placed in a supine position with the left knee in 90° of flexion. A 0.8  cm incision was made for entry on both sides of the horizontal patellar ligament to the articular cavity from the joint space of the left knee joint. An arthroscope was inserted from the lateral entry to examine the articular cavity orderly. During the exploration, the patellar articular surface of the knee joint was roughly distributed and seriously degenerated. The medial femoral condyle and the cartilage of the corresponding tibial articular surface were partially injured. More synovial folds were revealed in the lateral intersulcus. Synovial hyperplasia was found in the articular cavity. Numerous free rice‐like bodies were visible in the suprapatellar bursa and the intercondylar fossa. Probe examination revealed the presence of anterior and posterior cruciate ligaments. The lateral meniscus structure was fine, while the medial meniscus was worn and degenerative. The posterior external and posterior medial approaches were available and plenty of free rice bodies were explored in the posterior articular capsule. The remaining structures of the knee joints were intact. Tissues with synovial hyperplasia were removed and the cartilage was separated. The free bodies in the suprapatellar bursa, intercondylar fossa, and posterior joint bursa were removed. The articular cavity was rinsed with a large amount of normal saline and withdrew the arthroscopic devices following the presence of all devices was counted. The skin was sutured layer by layer, and ropivacaine and sodium hyaluronate were injected into the articular cavity after anastomosis. An elastic bandage was applied to the lower limb and the patient was transferred to PACU until recovery from anesthesia (Fig. 4).

## Discussion

RB is a polymorphic, polished free rice‐like body that presents mainly in synovial fluid with a hyaline chondroid appearance. RBS is rare and secondary to chronic arthritis due to different causes. Rheumatoid arthritis is more common. RBs are located in the synovial capsule of the affected joint, most commonly encountered in the knee and shoulder joints. Patients usually seek medical care due to chronic arthritis accompanied by painless masses.

The study of Popert *et al*. has indicated the presence of RB through detecting the synovial lavage fluid, which was collected from rheumatoid arthritis patients with chronic synovitis^6^. Some reports have also indicated that the formation of RB may be unrelated to the severity and imaging findings of arthritis. RB often locates in the articular cavity, periarticular bursa, and tendon sheath which is covered by synovial tissues. Some researchers believe that the formation of RB comes from synovial tissues^7^. Generally, the free bodies are similar to ceramic white rice grains. The mechanism of RB formation remains unclear, but it is likely to be an unusual complication of chronic bursitis. However, few pathological reports have been documented. There are several theories on RB formation: some have argued that intraarticular synovitis, microinfarction after ischemia, the subsequent synovium loss, and encapsulation by fibrin in synovial fluid are the most likely causes^8^. Others have suggested that RB has been initially formed in synovial fluid and increased with fibrin aggregation^6^, which is correlated with synovial fluid composition and viscosity^9^. Thickened synovium is usually manifested as chronic and nonspecific inflammation.

Identification with additional synovitis diseases is necessary before diagnosis, including rheumatoid synovitis, gouty synovitis, infectious synovitis, pigmented villonodular synovitis, and synovial chondromatosis^10^, ^11^, ^12^, ^13^, ^14^, ^15^. As the optimal detection of RBS, MRI findings are very typical. Through careful analysis, disease identification is possible among rheumatoid synovitis, gout synovitis, infectious synovitis, pigmented villonodular synovitis, and synovial chondromatosis. The pathological features of rheumatoid synovitis are synovial congestion, edema, and infiltration of a large quantity of monocytes, plasma cells, and lymphocytes. It is often accompanied by regional synovial cell necrosis, erosion and covered with cellulose‐like deposits. Pathological changes of gouty synovitis revealed that the course of gouty arthritis can be divided into an acute onset stage and a chronic tophi arthritis stage. Manifestations in the acute attack stage are a large number of visible polynucleate leukocytes infiltration in synovial membrane and synovia, and the presence of tiny urate crystals. As a result, an inflammatory reaction occurs namely synovial edema and congestion. Additionally, neutrophils aggregate abundantly. Pathological changes of infectious synovitis: synovitis is the first cause of joint infection, developed symptoms of synovial edema, congestion, and exudate. The course of infectious synovitis may be separated into three stages: the serous exudation phase, serous fibrin exudation phase, and purulent exudation phase. Pathological changes of synovial chondromatosis: generally, there are numerous blue‐gray cartilaginous nodules on the synovial surface of the joint, ranging in diameter from 2  mm to 1 cm. The nodules can fuse and form larger masses that block joint movement. Microscopically, cartilage nodules are composed of synovial connective tissue embedded in hyaline chondrocytes. It is easy to confirm the diagnosis by X‐ray examination in the clinic.

Taken together, patients with articular RB have few nodal symptoms at an early stage. Imaging MRI has a high soft tissue resolution and it is the first choice for RBS diagnosis^16^. In dilated synovial effusion, T2WI and fat suppression weighted nodular presented slightly high signals, T1WI low signals, and no enhancement after consolidation. The pathological report indicated multiple visible milky white tissues in hard texture with a total size of 7.5*2.5*2  cm. Histological results confirmed that the pathological center of RB was an amorphous acidophilic core, surrounded by collagen and fibrous tissues, with microvillous synovial hyperplasia in the bursa wall, infiltration of a large number of lymphoplasma cells, and minor giant cells. Under microscopy, thickened synovium is usually characterized by chronic, nonspecific inflammatory changes rather than pannus or granulomatous formation^17^. To date, the exact pathogenesis and optimal treatment of RB have not been reported. Further studies are necessary to fully recognize the pathogenesis and treatment of the disease.

### Conclusion

The RB synovitis of the knee joint is very rare in the clinic. As MRI examination can provide valuable information, clinicians should actively order MRI examination. Once the disease is diagnosed by examination, surgery is the optimal treatment.
